# Role of Siglec-7 in Apoptosis in Human Platelets

**DOI:** 10.1371/journal.pone.0106239

**Published:** 2014-09-17

**Authors:** Kim Anh Nguyen, Hind Hamzeh-Cognasse, Sabine Palle, Isabelle Anselme-Bertrand, Charles-Antoine Arthaud, Patricia Chavarin, Bruno Pozzetto, Olivier Garraud, Fabrice Cognasse

**Affiliations:** 1 GIMAP-EA3064, Université de Lyon, Saint-Etienne, France; 2 4D Multiphotonic Confocal Microscopy Platform (Hubert Curien Laboratory and UMR CNRS 5516), Université de Lyon, Saint-Etienne, France; 3 Centre de Microscopie Electronique Stéphanois - CMES–Saint Etienne, Université de Lyon, Saint-Etienne, France; 4 EFS Auvergne-Loire, Saint-Etienne, France; University of Kentucky, United States of America

## Abstract

**Background:**

Platelets participate in tissue repair and innate immune responses. Sialic acid-binding immunoglobulin-like lectins (Siglecs) are well-characterized I-type lectins, which control apoptosis.

**Methodology/Principal Findings:**

We characterized the expression of Siglec-7 in human platelets isolated from healthy volunteers using flow cytometry and confocal microscopy. Siglec-7 is primarily expressed on α granular membranes and colocalized with CD62P. Siglec-7 expression was increased upon platelet activation and correlated closely with CD62P expression. Cross-linking Siglec-7 with its ligand, ganglioside, resulted in platelet apoptosis without any significant effects on activation, aggregation, cell morphology by electron microscopy analysis or secretion. We show that ganglioside triggered four key pathways leading to apoptosis in human platelets: (i) mitochondrial inner transmembrane potential (ΔΨm) depolarization; (ii) elevated expression of pro-apoptotic Bax and Bak proteins with reduced expression of anti-apoptotic Bcl-2 protein; (iii) phosphatidylserine exposure and (iv), microparticle formation. Inhibition of NAPDH oxidase, PI3K, or PKC rescued platelets from apoptosis induced by Siglec-7 recruitment, suggesting that the platelet receptors P2Y1 and GPIIbIIIa are essential for ganglioside-induced platelet apoptosis.

**Conclusions/Significance:**

The present work characterizes the role of Siglec-7 and platelet receptors in regulating apoptosis and death. Because some platelet pathology involves apoptosis (idiopathic thrombocytopenic purpura and possibly storage lesions), Siglec-7 might be a molecular target for therapeutic intervention/prevention.

## Introduction

Beyond hemostasis, platelets have a variety of functions, particularly in innate immunity, host defense against infection, and inflammation, especially inflammatory processes such as atherosclerosis [1], [2]. We recently demonstrated that platelets can recycle a number of biological response modifiers (BRMs) [3], [4], [5], [6]. The list of platelet linked-BRMs has been tentatively described in several reports [7], [8]. One of the most important BRMs relative to platelet physiopathology is CD40-ligand (CD40L), which is present either associated with membranes or as a secreted, soluble form [9], [10]. Several recent data, including our own, demonstrated that platelets could sense external signals (such as pathogen-associated molecular patterns; PAMPs) differentially through a single type of pathogen recognition receptor. Upon PAMP recognition, platelets can influence the innate immune response appropriately for pathogens exhibiting different types of ‘danger’ signals by secreting a number of cytokines/chemokines or related receptors [2], [11]. Moreover, platelets can also sense danger signals such as damage-associated molecular patterns (DAMPs), also known as Alarmins, of which the family of sialic acid-binding immunoglobulin-like lectin (Siglec) belongs.

Siglecs are the best characterized as cell membrane receptor, I-type lectins, a subset of the immunoglobulin (Ig) superfamily that contains a sialic acid binding site in the amino-terminal Ig-like domain, which mediates sialic acid recognition [12]. The human Siglec family includes 14 members [13]. The function of Siglec molecules might explain the missing mechanistic link between cancer hypersialylation and immunological inhibition [14], [15]. Several Siglecs are restricted to expression in a distinct subset of leukocytes, and though all Siglecs bind glycans containing sialic acid, they differ in their recognition of the linkage biochemistry and spatial distribution [16], [17]. Siglecs contain a cytosolic immunoreceptor tyrosine-based inhibitory motif (ITIM), which recruits SH2-containing tyrosine phosphatases to the site of activation and halts the kinase phosphorylation cascade [15]. Several studies have shown that CD33-receptors trigger the release of anti-inflammatory cytokines, induce apoptosis, and inhibit cellular proliferation [18].

For many years, programmed cell death (apoptosis), was attributed exclusively to nucleated cells. However, apoptosis in platelets has been well described for 15 years [19]. Since platelets are not nucleated, describing their programmed death as apoptosis should be taken with caution. Leytin's group [20] clearly described all events of platelet death as apoptosis, but Jackson et al., [21] uses the term senescent platelet death. All events of activation-associated death are necrosis, since activation-associated platelet death results in increased inflammatory receptors, release of inflammatory mediators and aggregation that causes immune reactions. Because platelets are anucleate, their apoptosis leading to cell death is intriguing [20]. Two main pathways were reported—i) intrinsic and ii) extrinsic—that are highly regulated by intra-platelet signaling mechanisms [20], [22]. Furthermore, platelet apoptosis might play a role in hemostasis, thrombosis and inflammatory processes [22], [23].

Here we report the novel expression and localization of Siglec-7 in the α-granules of human platelets, and provide evidence that Siglec-7 regulates intrinsic apoptosis pathway in platelets.

## Materials and Methods

Informed consent of all donors was obtained prior to blood collection by the Regional Blood Service (EFS Auvergne-Loire: http://www.dondusang.net/rewrite/heading/810/efs/l-efs-en-regions/auvergne-loire.htm?idRubrique=810). A detailed and expanded ‘Material and Methods’ section is available in File S1.

### Platelet marker analysis

Platelet-rich plasma (PRPs) were prepared, as described in File S1. To test for Siglec-7 expression, platelets were incubated with 10 µg/ml of a polyclonal Siglec-7 antibody (pAb) reported to mediate neutralizing activities at 37°C for 30 min [24]. Platelets were then stimulated with a human disialoganglioside (GD2) (5 µg/ml, in 2∶1 chloroform:methanol, 200 µL) or a “control vehicle”. Alternatively, platelets were stimulated with 30 µm A23187 (a positive control for platelet apoptosis [20]), at room temperature (RT) for 30 min. Samples were washed twice with 1×PBS. Cells lysates were prepared by using cell extraction kit (Active Motif, La Hulpe, Belgium) following the manufacturer's instructions. Cytosolic proteins were quantified as described in File S1.

### Platelet scanning by electron microscopy

Platelets were stimulated with disialoganglioside (GD2), vehicle control, or A23187, as described above. Stimulated samples were fixed with 2.5% glutaraldehyde. After 30 min, platelets were washed twice with sodium cacodylate buffer and twice with water. Following dehydration, cells were sputter-coated with gold-palladium using Hummer-VI Sputtering Polaron SC7620 System (Anatech, Union City, CA, USA). Electron microscopy analysis was performed on a Hitachi scanning electron microscope, S3000N (Hitachi, Bron, France) at an accelerated voltage of 12 kV and working distance of 7.3 mM.

### Platelet marker analysis by confocal microscopy

#### Protein staining

Unless otherwise mentioned, all mAbs were purchased from Abcam. Immunostaining was performed as previously described and detailed in File S1 [25].

#### Confocal microscopy: image acquisition and analyses

Samples were examined with the Leica TCS-SP2 confocal scanning laser inverted microscope (Leica-Microsystem, Heidelberg, Germany), and image stacks were analyzed and processed using ImageJ software as detailed in File S1 [25].

### Analyses of the intracellular compartments

The list of reagents and solutions used in the present analyses of the intracellular compartments is given in File S1.

### Death and apoptosis marker analysis

Apoptosis was measured using a previously described method [26].

#### Platelet counts

A Coulter LH-500 hematology analyzer (Beckman Coulter, Villepinte, France) was used to count platelet numbers. Pretreatment counts were used to establish the absolute reference value (100%) for each sample. PRP samples (300 µL) were incubated with or without blocking anti Siglec-7 pAb, followed by treatment with Siglec-7 ligand, GD2, vehicle control, or A23187 [22].

#### Analyses of mitochondrial membrane potential (ΔΨm)

To measure mitochondrial membrane potential (Δ*Ψm*), following stimulation, a suspension of 5×10^6^ platelets in 100 µL was incubated with 40 nm DiOC6(3), a cell-penetrable green-fluorescent cationic dye (Invitrogen, Saint Aubin, France), for 30 min at RT. The samples were diluted to 500 µL in 1×PBS and analyzed by flow cytometry. Depolarization of mitochondria results in a decrease in the mean fluorescence intensity of platelet-bound DiOC6(3) in FL1.

#### Quantification of platelet microparticle assay

To induce apoptosis, PRPs were treated with A23187 (30 µM for 30 min at RT) or Siglec-7 ligand, GD2 (5 µg/ml for 30 min at RT). Both the treated and untreated platelets were blocked using anti-Siglec-7 pAb (10 µg/ml) for 30 min at 37°C. PRPs were centrifuged at 1000 ×*g* for 10 min. The supernatant was separated and centrifuged for 2 min at 13,000 ×*g* at RT. The resulting supernatant was collected and tested for platelet microparticles (PMPs). PMPs with the exposed PS surface were quantified using the Zymuphen MP-Activity kit (Hyphen Biomed Neuville-Sur-Oise, France) according to the manufacturer's instructions. Results were normalized to total platelet number (nM/10^7^ platelets) [27].

### Statistical analyses

Comparison of two means was performed by Student's *t*-test, except in PMP assays, where differences between two concentrations (median) were assessed using the Wilcoxon paired test. Analysis of variance was used to analyze the differences between group means in multifactor analysis (n≥3). A *p*-value <0.05 was considered statistically significant.

Correlation between two variables was assessed by Pearson's coefficient. The *p*-values were calculated to test the null hypothesis (Ho) that Pearson coefficient was not significantly different from zero; one given correlation was considered significantly different from zero, when *p*-values were less than the significance level α = 0.05.

## Results

### Human platelets express Siglec-7

**Figure 1:**
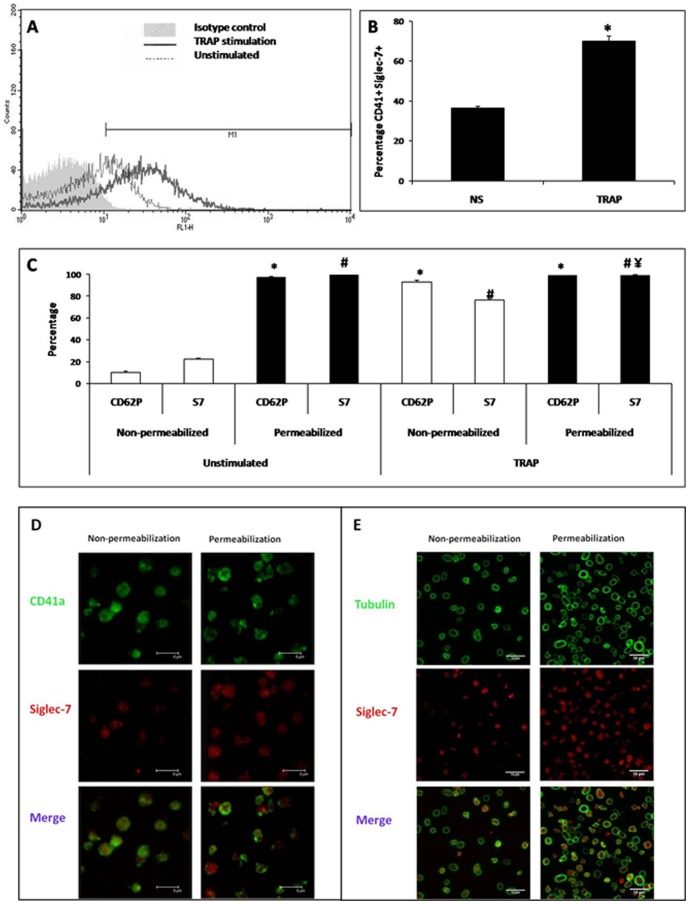
Expression of Siglec-7 in human platelets. (**A**), (**B**) Siglec-7 expression in platelets was analyzed by flow cytometry (n = 20). Platelets (stimulated with TRAP or vehicle control) were labeled with anti-CD41a and anti-Siglec-7 mAbs. Membrane expression of Siglec-7^+^ was increased after TRAP-induced platelet activation (*significant difference, of %CD41a^+^Siglec-7^+^ between TRAP-induced platelets activation *vs* resting platelets, *t*-test, *p*<0.05). (**C**) Membrane and intracellular localization of Siglec-7 in platelets. Platelets before and after permeabilization (stimulated with TRAP or vehicle control) were labeled with anti-CD41a, anti-CD62P (positive control for permeabilized platelets) and anti-Siglec-7 mAbs. Gating on the CD41a^+^ population, intra-platelet expression of Siglec-7 is significantly higher than its membrane expression (in both stimulated and unstimulated) (*, # significant difference (*t*-test, *p*<0.05) between %CD41a^+^CD62P^+^ or %CD41a^+^Siglec-7^+^, respectively, *vs* non-permeabilized resting platelets. (**D**) Distribution of Siglec-7 in platelets analyzed by confocal microscopy. Immunofluorescence labeling with anti–CD41a, and anti-Siglec-7 mAbs, and overlay (top to bottom). Siglec-7 staining in permeabilized (right panel) and non-permeabilized platelets (left panel) shows intracellular expression is more important than membrane expression. Scale bars = 6 µm. (**E**) Labeling with anti-tubulin and anti Siglec-7 antibodies and overlay (top to bottom). Siglec-7 is expressed on platelet membranes and tubulin is stained in permeabilized platelets. Tubulin was labeled to demarcate platelet borders and Siglec-7 is mostly observed in intracellular compartments. Scale bars = 10 µm.

Expression of Siglec-7 in resting human platelets was analyzed by flow cytometry. Fig. 1A shows representative flow cytometry histogram profiles for Siglec-7 expression. A mean of 36±0.84% of CD41a^+^ platelets expressed Siglec-7 in the absence of deliberate stimuli. Following stimulation with TRAP, there was an increase in Siglec-7 expression of 70±2.44%, indicating that platelet stimulation resulted in mobilization of Siglec-7 (*p*<0.05, n = 20) (Fig. 1B). Almost all permeabilized and unstimulated (resting) platelets showed expression of either intracellular and/or membrane-bound Siglec-7 (99.66±0.07% and 22.7±0.97% respectively; *p*<0.05), as well as CD62P (97.7±0.42% and 9.9±1.1%, respectively; *p*<0.05). There was no gain following TRAP stimulation (Fig. 1C).

Absence of CD3-T, CD19-B cells, and CD14-monocytes was confirmed by flow cytometry, suggesting no contaminated leukocytes in the platelet preparations (Fig. S5 in File S1).

As flow cytometry performed on permeabilized platelets does not delineate surface and intracellular protein expression, we examined the intracellular localization of Siglec-7 using confocal microscopy. Platelets, before and after permeabilization, were plated on poly-L-lysine coated glass cover slips, and stained with Siglec-7 mAb and CD41a or Tubulin (Fig. 1D,E). Confocal microscopy revealed that Siglec-7 is mostly present in the intracellular compartment of human platelets.

**Figure 2:**
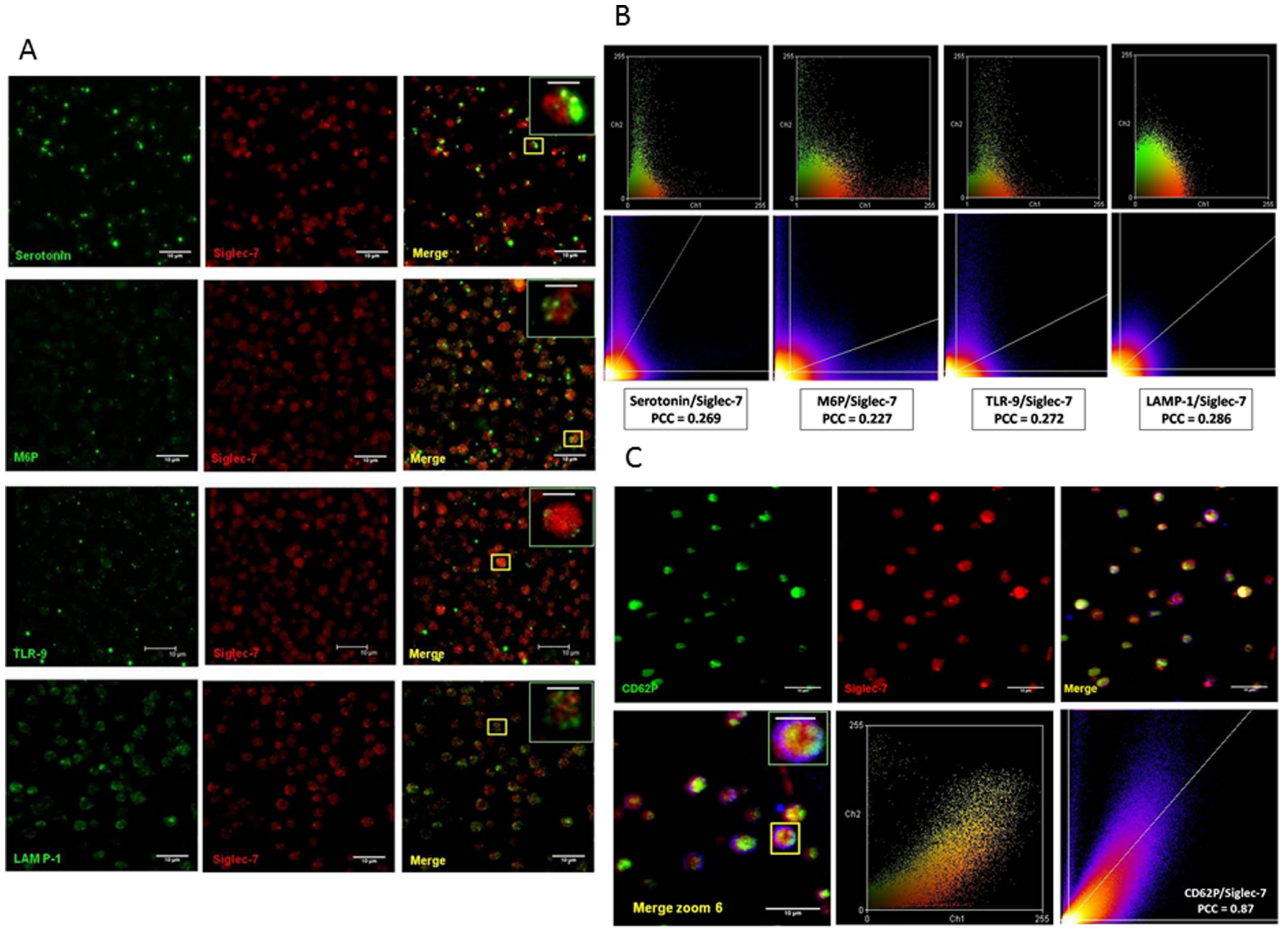
Siglec-7 localization in human platelets. (**A**) Siglec-7 does not colocalize with markers for dense granules, lysosomes, T-granules or endosomes. From top to bottom: intra-platelet Siglec-7 was co-stained with serotonin (dense granules), M6P (endosomes), TLR-9 (T-granules), and LAMP-1 (lysosomes) using two distinct colored secondary antibodies. Insets represent magnified regions shown in yellow boxes. Scale bars = 10 µm, except insets where scale bars = 2 µm. (**B**) Scattergram, scatter plot, and colocalization analysis of Serotonin/Siglec-7; M6P/Siglec-7; TLR-9/Siglec-7; and LAMP-1/Siglec-7 (left to right). Siglec-7 did not colocalize with any proteins (Pearson correlation analysis, *p*>0.05). (**C**) Siglec-7 colocalized with CD62P, an α-granule marker. Scale bars = 10 µm except insets bars, which = 2 µm. Pearson correlation analysis, *p*<0.05.

To identify the precise intracellular localization of Siglec-7 further, platelets were permeabilized and co-stained with Abs for known marker proteins of different organelles: CD62P for α-granules; serotonin for dense granules; M6P for endosomes; TLR-9 for T-granules and LAMP-1 for lysosomes. Siglec-7 did not colocalize with serotonin [Pearson's correlation coefficient (PCC) = 0.269, *p*>0.05), M6P (PCC = 0.227, *p*>0.05), TLR-9 (PCC = 0.272, *p*>0.05), or LAMP-1 (PCC = 0.286, *p*>0.05) (Fig. 2A, from top to bottom and Fig.2B from left to right). However, Siglec-7 showed significant colocalization with the α-granule marker CD62P (PCC = 0.87, *p*<0.05) (Fig. 2C), suggesting Siglec-7 is predominantly localized to α-granules in platelets. Representative maximum Z projection images for Siglec-7 and CD62P (Fig. S1 in File S1) further confirmed that Siglec-7 is distributed in the same compartment as that of CD62P.

We also analyzed the expression profiles of other Siglec family proteins in human platelets. Among all the Siglec proteins tested, Siglec-7 appeared to be the highly abundant and most consistently expressed Siglec, whereas Siglec-9 and Siglec-10 had low levels of expression (Fig. S2A,B in File S1).

### Characterization of Siglec-7 as a marker for platelet activation

**Figure 3:**
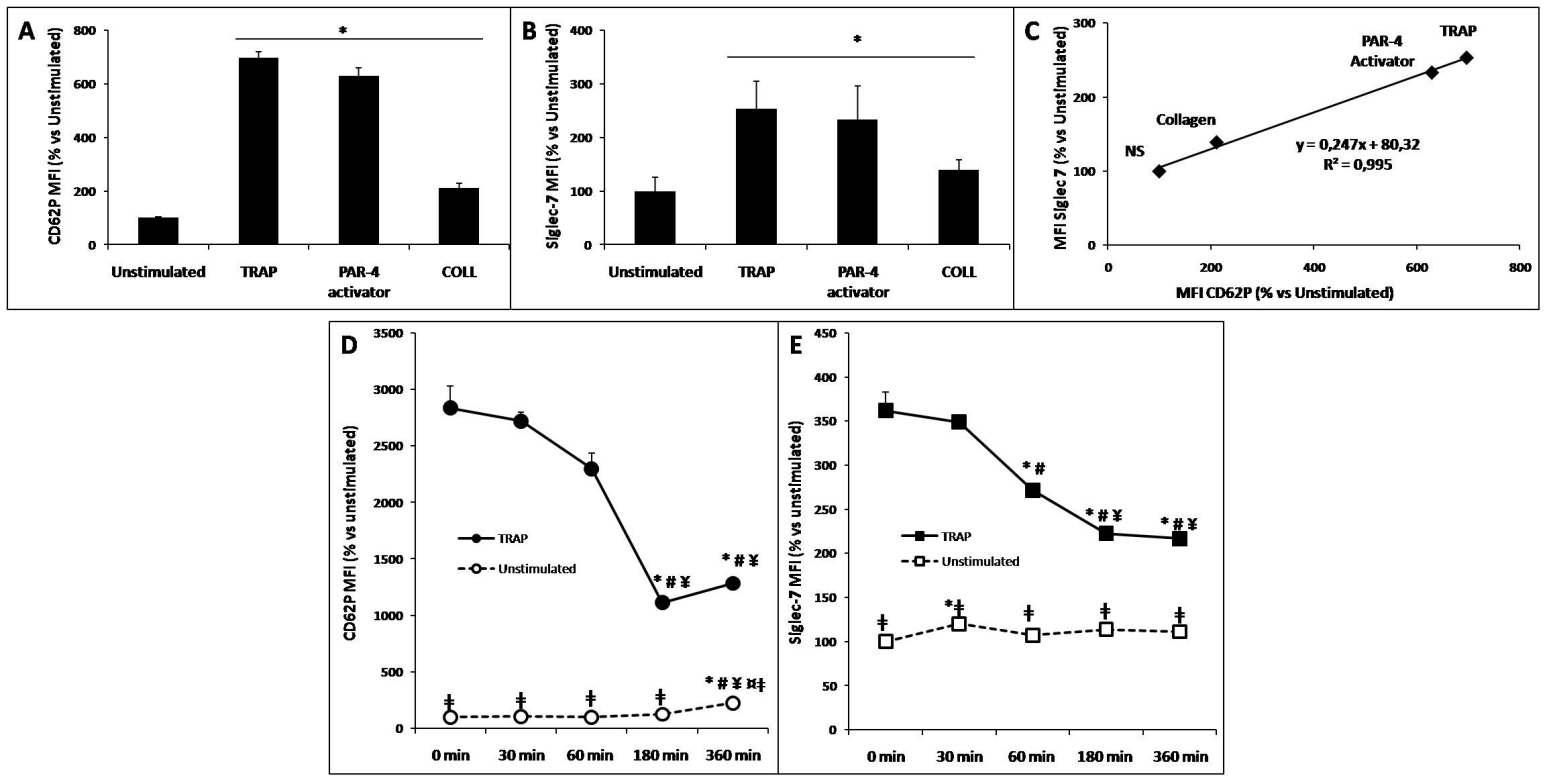
Characterization of Siglec-7 as a novel marker of platelet activation. (**A**),(**C**). Agonist-induced platelet activation (n = 10). Platelets were stimulated with different agonists: TRAP (PAR-1), PAR-4 activator, collagen (β_2_α_1_ integrin and GPVI-FcR gamma). Membrane MFI expression of CD62P (**A**) and Siglec-7 (**B**) was correlated under all experimental conditions (PCC = 0.995, *p* = 0.002) (**C**). (**D**),(**E**). Kinetics of CD62P (**D**) and Siglec-7 (**E**) expression on platelet membranes (n = 5). Membrane expression of Siglec-7 and CD62P in platelets under un-stimulated and TRAP stimulated conditions was analyzed by flow cytometry. MFI changes between CD62P and Siglec-7 were correlated over time (unstimulated: PCC = 0.842; *p* = 0.016; stimulated by TRAP: PCC = 0.958; *p* = 0.01). *, #, ¥, ¤: significant differences (analysis of variance, *p*<0.05) between MFI of Siglec-7 or CD62P marker over time *vs* 0, 30, 60, and 180 min respectively; ‡: *t*-test, *p*<0.05) MFI of Siglec-7 or CD62P marker in TRAP-induced platelets activation *vs* resting platelets (expression of CD62P and Siglec-7 in MFI was reported for unstimulated conditions at 0 min, which was considered as 100%).

Activated platelets from PRPs were analyzed and quantified for the activation marker CD62P, and for Siglec-7, using flow cytometry (n = 10). Platelet responses upon stimulation with TRAP, PAR-4 activator and collagen (5 µM, 200 µM and 50 µg/ml, respectively) were measured as the mean fluorescence index (MFI, m ± SEM). The MFI for CD62P on unstimulated platelets was 13.52±0.60. TRAP treatment resulted in increased MFI (94.10±3.04%, *p*<0.05), PAR-4 activator increased the MFI to 85±4 (*p*<0.05), and collagen increased it to 28.56±2.20 (*p*<0.05) (Fig. 3A). Similar to that for CD62P, following stimulation, Siglec-7 induced a significant increase in MFI. The baseline MFI in unstimulated cells was 301.59±76.46 and TRAP stimulation increased the MFI to 764.78±155.00 (*p*<0.05). Stimulation with the PAR4 activator (Ala-Tyr-Pro-Gly-Lys-Phe-NH2, Sigma Aldrich) resulted in an MFI of 704.15±189.50 (*p*<0.05); and collagen stimulation increased the MFI to 419.78±58.30 (*p*<0.05) (Fig. 3B). There was a significant correlation between Siglec-7 and CD62P MFI (PCC = 0.995, *p*<0.05) (Fig. 3C). Additionally, there was a close correlation between the expression of CD62P and Siglec-7 over time on the membrane surface of unstimulated (PCC = 0.842, *p* = 0.016) and TRAP stimulated platelets (PCC = 0.958, *p* = 0.01). In TRAP stimulated platelets, the activation marker protein started to decline steadily after reaching peak expression at t0 (data not shown). This is probably due to the decay of the surface protein or cleavage of the membrane bound protein to the soluble form. This mechanism was previously described for CD62P [28]. To determine whether Siglec-7 also showed similar cleavage and translocation from platelet membranes, we tested levels of soluble Siglec-7 in TRAP treated platelets at different time points. Incubation of platelets with TRAP (30 min) resulted in small, but, insignificant changes (*p* = 0.9): soluble Siglec-7 secretion (3820.1 pg/2×10^8^ to 3982.3 pg/2×10^8^ platelets/ml). Furthermore, there was no change in secretion levels up to 6 h in both the unstimulated- and TRAP-stimulated platelets (Fig. S3 in File S1).

We next determined whether the reduced surface expression of Siglec-7 and CD62P was due to degradation by matrix metalloproteinases (MMPs), and tested platelets for MMP expression levels. There was no significant correlation between the expression of MMP 1, 2, and 9, and Siglec-7 and CD62P in the corresponding supernatants (Fig.S4 in File S1), suggesting MMPs are not involved in cleavage and/or degradation of Siglec-7 on platelet surface membranes.

Together, our results suggest that Siglec-7 expression is similar to that of the activation marker CD62P, indicating a close relationship between Siglec-7 and platelet activation.

### Effect of Siglec-7 ligand binding on platelet activation

**Figure 4:**
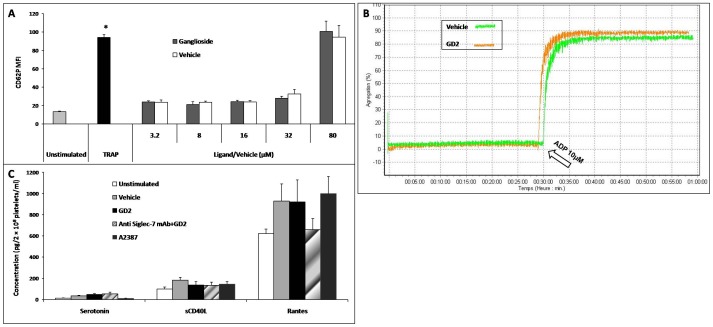
Effects of Siglec-7 ligand, ganglioside, on platelet function. (**A**) Platelet activation upon stimulation with GD2 (n = 10). *: Significant difference, *t*-test, *p*<0.05, changes in membrane expression of CD62P in platelets stimulated by TRAP *vs* resting platelets. There was no significant change in the expression of CD62P between platelets stimulated with GD2 or vehicle control (*t*-test, *p*>0.05). Similar results were observed for GD3 and GT1b stimuli. Thus, gangliosides show no effect on platelet activation. (**B**) Platelet aggregation (n = 3) analyses. Incubation of platelets with either GD2 or vehicle did not induce platelet aggregation or alter their response to ADP stimulation (10 µM). (**C**) Platelet secretion analysis: Platelet secretion of serotonin, sCD40L, and RANTES induced by GD2 stimulation (n = 10). The secretion of resting platelets was slightly lower than both the vehicle and GD2-stimulated platelets; however, there was no significant difference between the latter two groups (*t*-test, *p*>0.05).

We next examined whether exposure of platelets to GD2, a Siglec-7 ligand, altered platelet activation by analyzing CD62P and PAC-1 expression (Fig. 4A) and aggregation (Fig. 4B). GD2 is soluble in a chloroform:methanol mix of 2∶1. The mixture alone was used as the vehicle control. GD2 (3.2 to 80 µM) did not induce a statistically significant elevation in CD62P expression levels compared with the vehicle control. Similar to CD62P expression, PAC-1 binding (an indication of expression of the fibrinogen binding domain of the αIIβ3 integrin, data not shown) was unaffected by GD2 (80 µM) or vehicle (data not shown).

Furthermore, neither GD2 nor the vehicle control-treated platelets showed aggregation over 30 min. When ADP (10 µM) was added to PRP, after 30 min of GD2 or vehicle treatment, there was a submaximal platelet aggregation. Similar results were observed with another agonist (TRAP) used at two concentrations (5 and 10 µM) (data not shown).

These results suggest that while GD2 itself is not capable of activating platelets, it does not prevent ADP-induced aggregation. In addition, GD2 did not result in significant changes in soluble factor secretion, compared with vehicle control (Fig. 4C). Thus, despite Siglec-7 being expressed and functional in platelets, our data show that it is not essential for mediating hemostasis or immunity/inflammation/repair, the main two key functions of platelets.

### Siglec-7 cross-linking induces apoptosis and death in platelets

**Figure 5:**
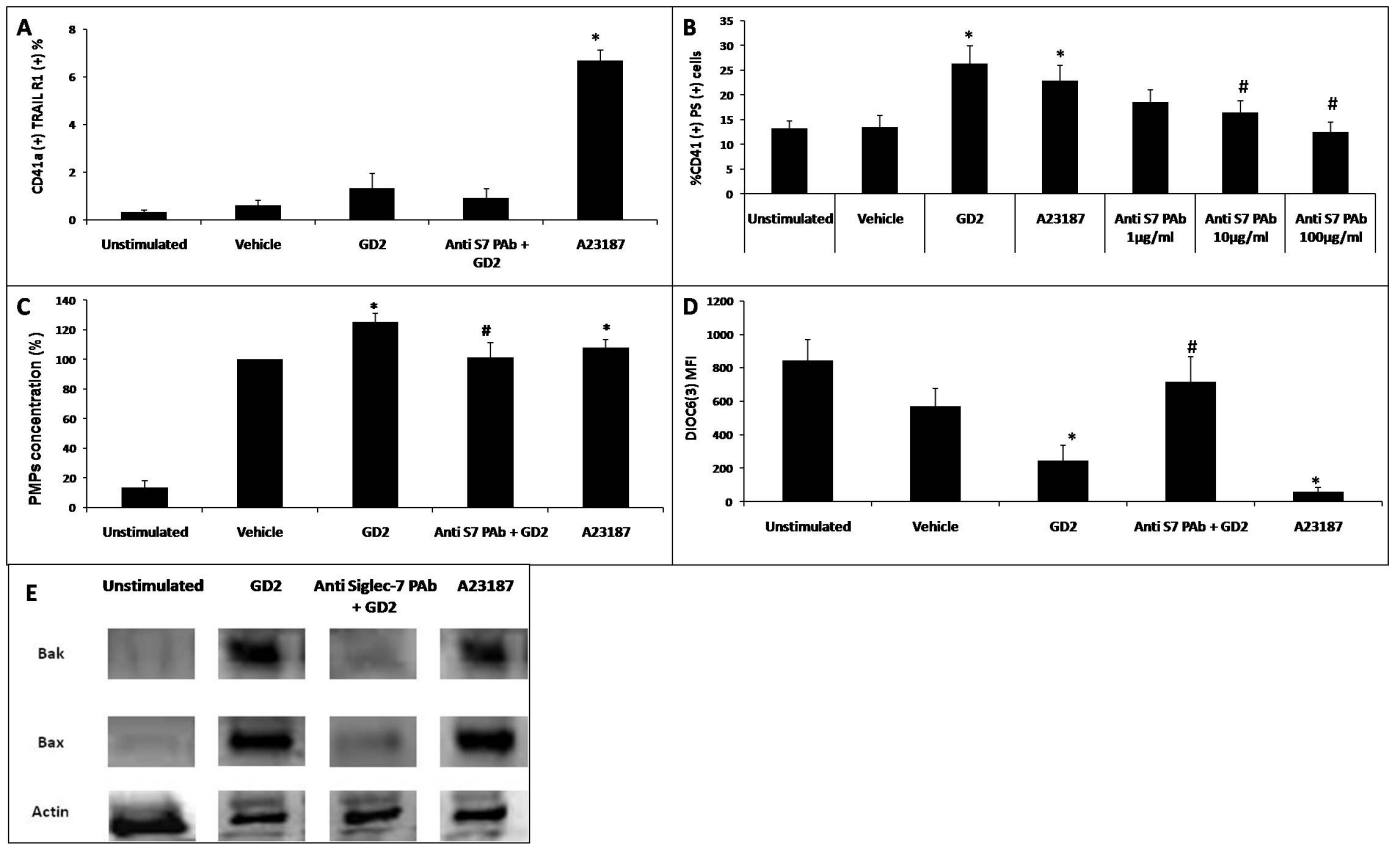
Effect of GD2 on platelet death. (**A**) Cell death induced by GD2 stimulation. Platelets untreated or pre-treated with blocking anti-Siglec-7 pAb were incubated with vehicle or GD2. A23187 was used as a positive control for apoptosis. Cell death rate (%) was measured as the percentage of diminution in platelet counts relative to unstimulated platelets. *, #: significant differences (*t*-test, *p*<0.05) *vs* vehicle or GD2 stimulated conditions. Results are representative of five independent experiments. (**B**)(**C**) Morphology of platelets analyzed by scanning electron microscopy. (**B**) The morphology of platelets stimulated with GD2 resembled apoptotic cells as characterized by blebbing, filopod extrusion (1,2) and cell shrinkage (3). (**C**) Resting platelets have a discoid shape with a smooth cell surface. (**D**): A23187-induced platelet apoptosis, red (Scale bars = 20 µm) and green (Scale bars = 10 µm) arrows that represent platelet apoptosis.

Loss of platelets was observed during *in vitro* experiments. This prompted us to examine the role of Siglec-7 in platelet apoptosis following its engagement to the surface of platelets upon cross-linking with GD2. GD2 induced a significant increase in platelet loss/death compared with the vehicle treated cells (29.88±4.97% vs 12.00±3.21%); A23187 induced platelet loss/death by 68.40±0.34% (Fig. 5A). Furthermore, GD2-induced platelet death was significantly attenuated (29.88±4.97% vs 12.69±3.89%; *p*<0.05) when platelets were pre-incubated with neutralizing anti-Siglec-7 pAb for 30 min at 37°C (Fig. 5A). Electron microscopy analysis of GD2 and vehicle treated platelets (Fig. 5B,C) revealed morphological changes closely resembling filopod and lamellipod extrusion signs of platelet activation, but also typical apoptosis profiles in GD2 treated platelets, including blebbing [20] (Fig. 5B.1,2) and cell shrinkage (Fig. 5B.1,3). The morphology of GD2 treated platelets is similar to that of A23187 treated platelets (Fig. 5D). These morphologies are comparable to those reported to occur in platelet apoptosis induced by high shear stress [29]. Thus, engagement of Siglec-7 by its ligand, GD2, showed an increase in platelet loss, displaying features of cell death and apoptosis. To determine whether this observation relates to apoptosis, we sought to explore various apoptosis pathways in platelets in relation to the Siglec-7::GD2 ligand interaction.

Three main pathways are responsible for platelet apoptosis [26]: i) extrinsic pathway, initiated by interactions between the TNF superfamily death ligands and the cell surface TNF receptors such as TRAIL receptor; ii) intrinsic pathway, characterized by the expression of pro-apoptotic and anti-apoptotic Bcl-2 family proteins, Bak, Bax, and depolarization of the mitochondrial inner membrane potential; and iii) cellular pathway, characterized by the exposure of PS, the activation of apoptosis executioner caspase-3, and PMP formation.

**Figure 6:**
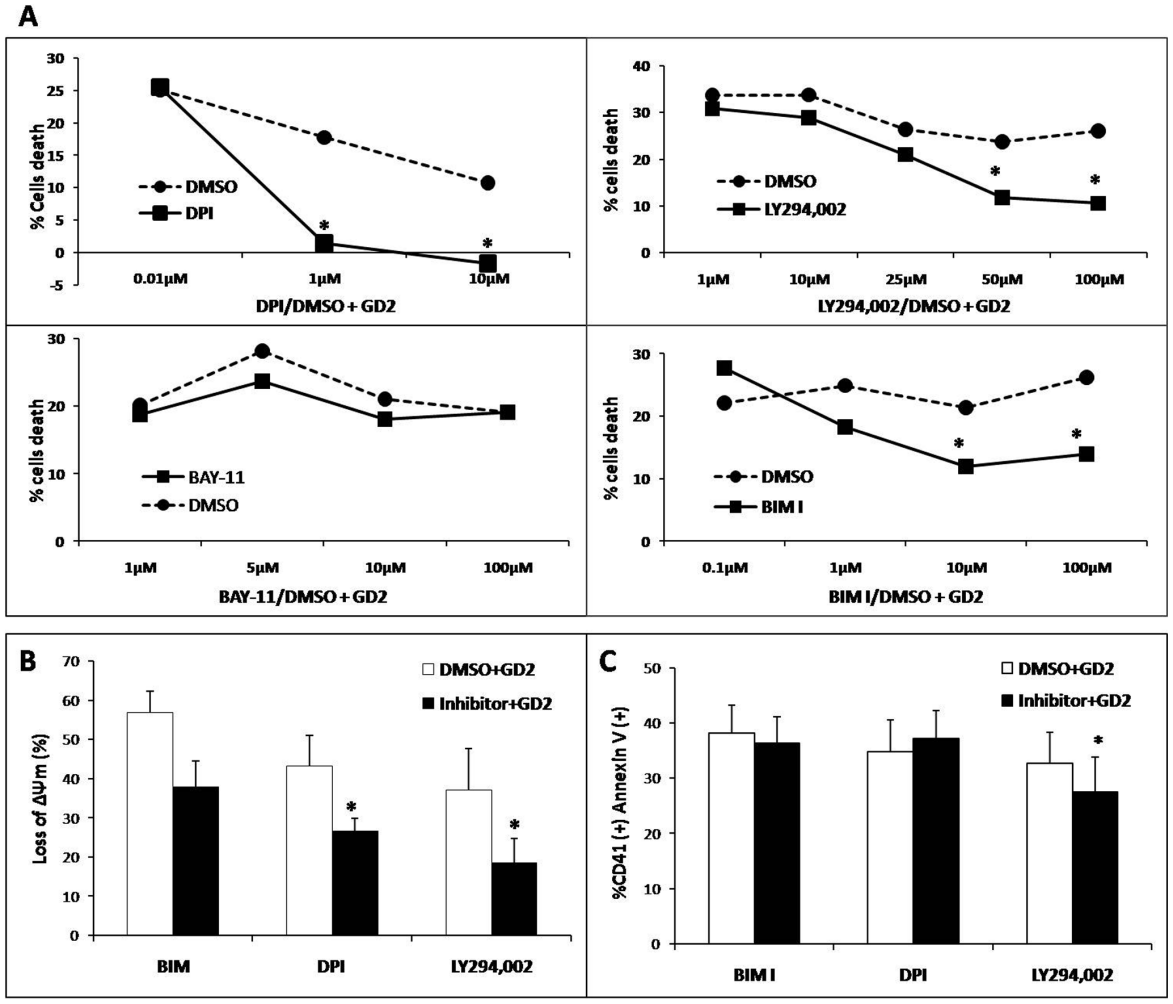
Mechanism of GD2-induced platelet apoptosis. Both platelets pretreated with blocking anti-Siglec-7 PAb and untreated platelets were incubated with vehicle or GD2. A23187 was used as an apoptosis positive control. Different parameters of apoptosis pathways in platelets were analyzed. (**A**) Expression of TRAIL R1 in platelets stimulated with GD2 (n = 12). *: significant difference (*t*-test, *p*<0.05) *vs* resting platelets. The extrinsic pathway may not be involved in platelet apoptosis induced by GD2. (**B**) Analyses of phosphatidylserine (PS) exposure (n = 13). The extent of PS exposure by GD2 was reduced by blocking anti-Siglec-7 pAb in a concentration-dependent manner. *, #: Significant difference (*t*-test, *p*<0.05) *vs* vehicle or GD2 stimulated conditions respectively. (**C**) Platelet microparticle assay (n = 10). PMP formation was calculated with respect to the concentration in vehicle conditions (arbitrarily designated 100%). *, #: significant difference (Wilcoxon paired test, *p*<0.05) *vs* vehicle or GD2 stimulated conditions, respectively. (**D**) ΔΨm depolarization. ΔΨm depolarization resulted in decreased DIOC6(3) accumulation. *, #: Significant difference (*t*-test, *p*<0.05) *vs* vehicle or GD2 stimulated conditions, respectively. Results are representative of five independent experiments. GD2-induced mitochondrial depolarization in platelets treated with anti-FcγRII mAb or PBS control. *, ** significant difference (*t*-test, *p*<0.05) of ΔΨm between GD2 stimulated platelets vs unstimulated platelets in the presence or absence of anti-FcγRII mAb. #, ¥: Anti-Siglec-7 PAb + GD2 vs only-GD2 stimulated platelets in the presence or absence of anti-FcγRII mAb (n = 5). NS: Not significant. (**E**) Western blot demonstrates strong expression of Bax and Bak in GD2-treated human platelets and this expression was prevented by blocking anti Siglec-7 pAb. Results are representative of three independent experiments.

We first explored the extrinsic apoptosis pathway by measuring whether platelets expressed a death receptor, i.e. TRAIL-R1 after engagement of Siglec-7 by GD2 (compared with stimulation with A23187) (Fig. 6A). Resting platelets showed a weak expression of TRAIL-R1 (0.33±0.08%), which varied minimally after stimulation with either vehicle or GD2 (0.62±0.20% and 1.30±0.62%, respectively; not significant), contrary to control A23187 group, which induced a significant, though modest, increase in TRAIL-R1 expression in platelets (6.70±0.43%; *p*<0.05).

We next explored the extra-mitochondria cellular pathway and examined whether platelets expose PS following Siglec-7 engagement by GD2 or vehicle control. GD2 stimulated platelets showed a significant increase in expression of PS compared with that of vehicle or A23187 treatments (26.35±3.6%, 22.8±3.15%, and 13.47±2.44% respectively; *p*<0.05) (Fig. 6B). However, no significant difference in PS expression was observed (16.45±2.33%) when platelets were pre-incubated with varying concentrations of neutralizing anti-Siglec-7 pAb. Comparable data were observed regarding PMP formation; PMPs were significantly increased upon GD2 treatment, but not affected by anti-Siglec-7 pAb (Fig. 6C).

We finally explored the mitochondria-dependent apoptosis pathway (the intrinsic pathway). We used DiOC6(3) (3,3′-dihexyloxacarbocyanineIodine), a cell-penetrating green-fluorescing cationic dye, to determine the effect of GD2 on mitochondrial membrane potentials. As illustrated in Fig. 6D, exposure of platelets to GD2 resulted in a decline of the mitochondrial potential; the baseline DiOC6(3) MFI decreased from 846.2 to 245±89.6 (GD2; *p*<0.05). The vehicle treated platelets did not show a statistical alteration in MFI (571±106). However, A23187 treatment almost completely abolished the membrane potential (59.1±26.0; *p*<0.05). Of note, the mitochondrial potential was restored when platelets were pre-incubated with pAb to Siglec7, prior to GD2 treatment (716.40±151.13 *vs* 245.0±89.6; *p*<0.05). Similar changes were observed in the expression levels of the pro-apoptotic Bcl-2 family proteins (Bak, Bax) that also characterize the mitochondrial apoptosis pathway (Fig. 6E), when the following protocol was used: PRPs were treated with A23187 (30 µM for 30 min at RT) or Siglec-7 ligand, GD2 (5 µg/ml for 30 min at RT). Both the treated and untreated platelets were blocked using anti-Siglec-7 pAb (10 µg/ml) for 30 min at 37°C.

To further ensure that GD2 binding to Siglec-7 induced apoptosis in platelets, we used anti-Siglec-7 pAb or a control IgG, to rule out the role of FcγRII, using an anti-CD32 mAb. All Fcγ receptors (FcγR) belong to the immunoglobulin superfamily and are critical for inducing phagocytosis of opsonized (coated) microbes. Activation of FcγRIII by IgG promotes cell death by triggering apoptosis, and is thus coupled to the associated ITAM signaling [30]. As expected, none of the control IgGs had any effect on GD2-induced apoptosis in platelets (Fig. 6). Moreover, anti-CD32 mAb had no significant impact on GD2-triggered platelet mitochondrial depolarization or on protective effects induced by anti-Siglec-7 pAb (Fig. 6). Platelet exposure to GD2 was followed by a decline in mitochondrial membrane potential. In the presence of blocking anti-FcγRII using an anti-CD32 MAb, the loss of ΔΨm depolarization increased but was not statistically significant (Fig. 6). Our results are similar to those from Martini et al. [31], who showed that GD3 is expressed on the surface of platelets and internalized rapidly, where it transitorily associates with Src family tyrosine kinase, Lyn and the FcγR chain. This sequence of events ultimately leads to an enhanced CD32 expression on the platelet membrane.

Our results indicate that GD2-induced apoptosis in platelets involves both the extra-mitochondria cellular pathway and the intrinsic mitochondria-dependent pathway, and is independent of the extrinsic pathway. Neutralization with anti-Siglec-7 pAb can prevent apoptosis and cell death.

### Identification of key pathways in platelet signaling of Siglec-7 engagement

**Figure 7:**
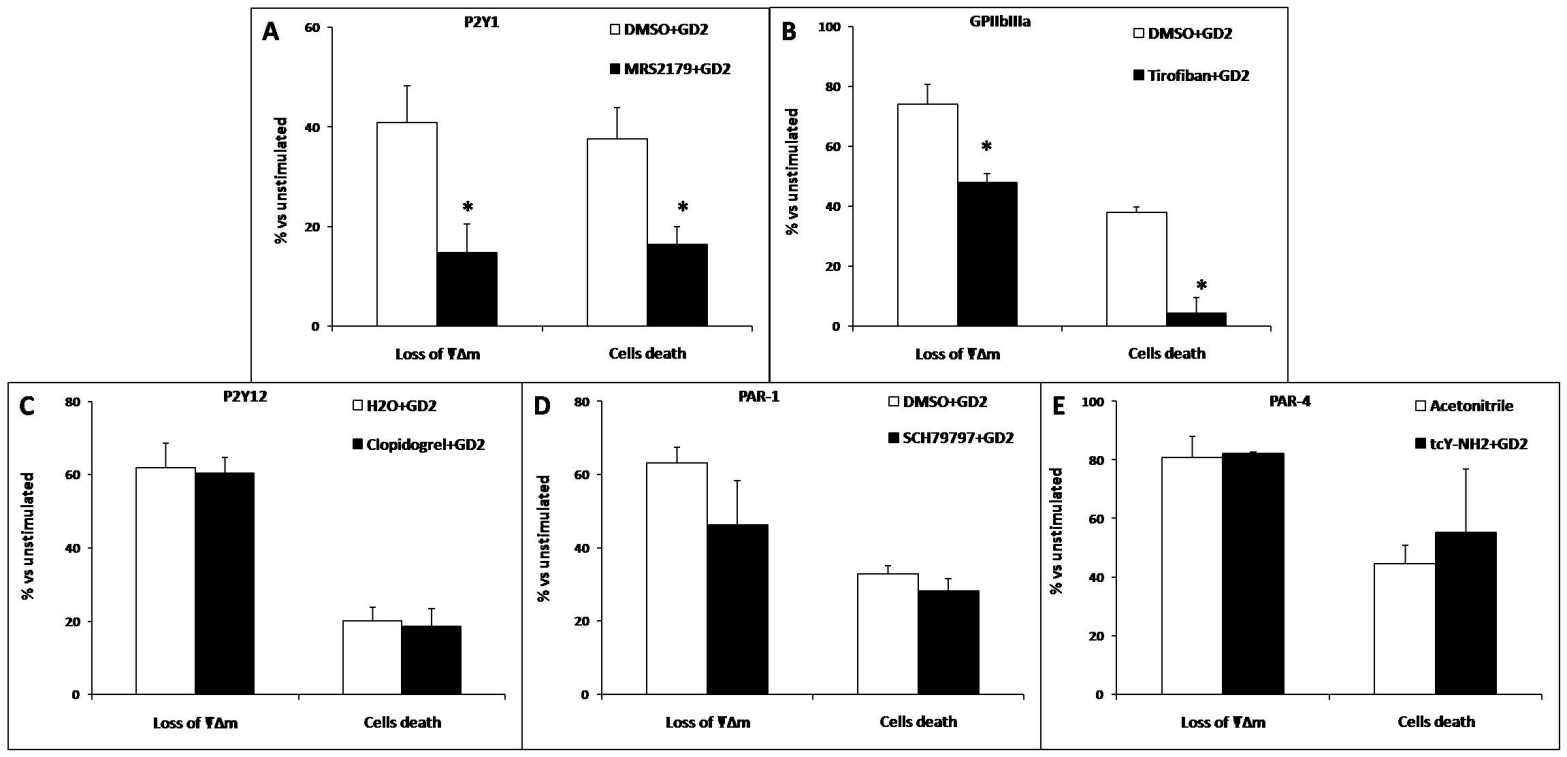
PI3K inhibitors reduce GD2-induced platelet apoptosis (n = 15). (**A**) Platelets were pre-treated with varying concentrations of intracellular pathway inhibitors (DPI for NAPDH oxidase, LY294,002 for PI3K, BIM I for PKC and BAY-11 for NFκB) or DMSO (negative control) followed by stimulation with GD2. Cell death rate (%) was calculated by the percentage of diminution in platelet number in comparison to unstimulated platelets. DPI, BIM I, and LY294,002 prevented cell death in a concentration-dependent manner. *: Significant difference (*t*-test, *p*<0.05) between inhibitor *vs* DMSO treated. The lowest concentration of inhibitors with a significant effect (DPI 1 µM, LY294,002 50 µM, BIM I 10 µM) (no difference at a higher concentration) was selected to treat platelets. (**B**) Loss of ΔΨm resulted in reduced accumulation of DIOC6(3), and was calculated as the percentage of diminution in DIOC6(3) MFI compared with unstimulated platelets. (**C**) Phosphatidylserine exposure (left panel: percentage of CD41a^+^, Annexin V^+^; right panel: MFI). *: Significant difference (*t*-test, *p*<0.05) between inhibitor *vs* DMSO pretreated platelets. Thus, PI3K inhibitor prevented both ΔΨm depolarization and PS exposure in platelets, while NAPDH oxidase inhibitor prevented only ΔΨm depolarization.

Prior to GD2 treatment, platelets were treated either with varying concentrations of intracellular pathway inhibitors (DPI for NAPDH oxidase; LY294,002 for PI3K; BIM I for PKC; and BAY-11 for NFκB), or dimethyl sulfoxide (DMSO) vehicle control. In this study, the concentration of DMSO vehicle control was similar to the concentration of DMSO used for diluting each intracellular pathway inhibitor. However, for DPI, the DMSO concentration was higher than for the other inhibitors (DPI 1/600; LY294 1/14,000; BAY11 1/10,000; and BIM I 1/2400). All experiments were independent and performed using cells from various donors. Therefore, we controlled for the effects of DMSO as much as possible. Inhibition of NAPDH oxidase (by DPI), PI3K (by LY294,002), and PKC (by BIM I) resulted in a significant reduction in platelet death in a dose-dependent manner. Significant differences in cell death were observed: DPI 1 µM (1.40% vs 17.75%, *p*<0.05), LY294,002 50 µM (11.74% vs 23.74%, *p*<0.05), BIM 10 µM (9.4% vs 25.6%, *p*<0.05) compared with DMSO treated platelets. Inhibition of NFκB with BAY-11 did not show any significant changes in platelet death (Fig. 7A). We measured changes in ΔΨm following treatment with the inhibitors. Specific inhibition of NAPDH oxidase (DPI, *p* = 0.037) or PI3K (LY294,002, *p* = 0.025), but not PKC (BIM I, *p* = 0.136) showed a significant increase in ΔΨm, further suggesting that these inhibitors protect platelets from GD2-induced apoptosis, in a mitochondria-dependent pathway compared with DMSO (mean ± SEM): 26.54±6.65% vs 43.21±5.38% (1.6-fold decrease) and 18.51±3.29% vs 37.04±7.80% (2-fold decrease) (*p*<0.05; Fig. 7B). Only the specific inhibition of PI3K by LY294,002, (*p* = 0.049) but not NAPDH oxidase (*p* = 0.161) or PKC (*p* = 0.938) significantly prevented the exposure of PS, an extra-mitochondria event of GD2-induced platelet apoptosis (27.51±2.51% *vs* 32.65±3.60% in control DMSO, *p*<0.05) (Fig. 7C).

Collectively, our results reveal that NAPDH oxidase and PI3K activities are responsible for platelet apoptosis induced by GD2.

### Cooperation between platelet hemostatic receptors and Siglec-7 to induce platelet apoptosis

Having shown that Siglec-7 is present on the surface of resting platelets and activated platelets, and is involved in controlling apoptosis, we next studied the effect of inhibition of platelet surface hemostatic receptors on Siglec-7-induced apoptosis and cell death.

**Figure 8:**
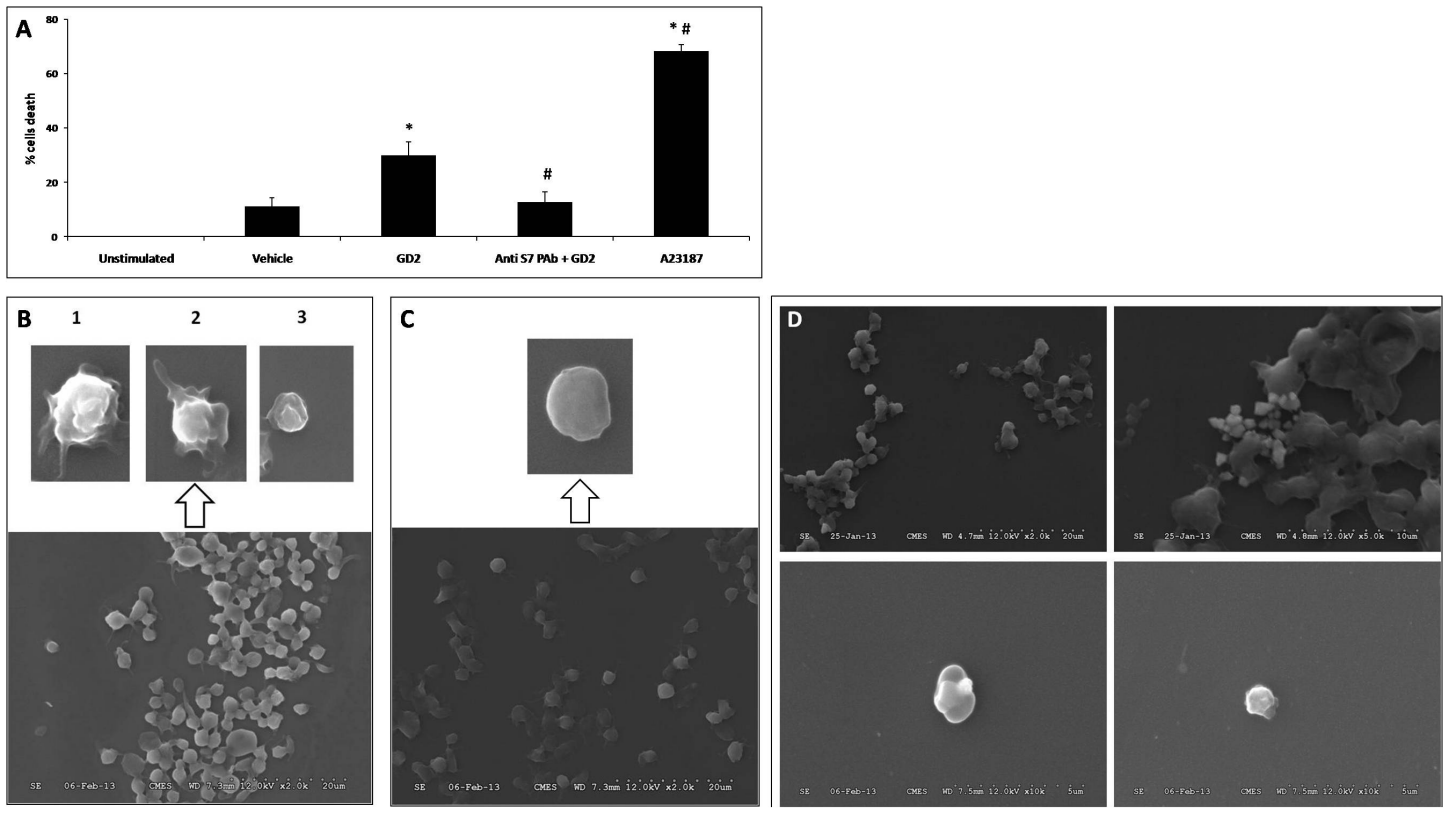
Ganglioside-induced platelet apoptosis is regulated by platelet P2Y1 and GPIIbIIIa antagonists (n = 10). Platelets were pre-treated with antagonists for the corresponding platelet receptors: (**A**) MRS2179 100 µM: P2Y1 antagonist; (**B**) Tirofiban 10 µM: GPIIbIIIa antagonist; (**C**). Clopidogrel 476 µM: P2Y12 antagonist; (**D**) SCH79797 10 µM: PAR-1 antagonist; (**E**) tcY-NH2 400 µM: PAR-4 antagonist) or DMSO at the same dilution (negative control) followed by stimulation with GD2. The cell death rate (percentage of diminution in platelet counts) and ΔΨm depolarization (percentage of diminution in DIOC6(3) MFI) were prevented by P2Y1 and GPIIbIIIa antagonists. * Significant difference (*t*-test, *p*<0.05) between antagonist *vs* DMSO pretreated platelets.

We investigated GD2 ganglioside-induced platelet apoptosis by treating platelets with similar concentrations of inhibitors against P2Y1, (MRS2179, 100 µM, Fig. 8A); GPIIbIIIa, (Tirofiban, 10 µM, Fig. 8B); P2Y12, (Clopidogrel, 476 µM, Fig. 8C); PAR-1, (SCH79797, 10 µM, Fig. 8D) and PAR-4, (tcY-NH2, 400 µM, Fig. 8E). DMSO was used as a negative control for all experiments. The platelet count and ΔΨm (decrease of DIOC6(3) accumulation) were measured. Platelets were first exposed to either inhibitors of the hemostatic receptors or DMSO, followed by GD2. Inhibition of P2Y12, PAR-1, and PAR-4 was ineffective for GD2-induced platelet death and ΔΨm depolarization. In contrast, inhibition of P2Y1 and GPIIBIIIa was associated with reversed GD2-induced cell death and ΔΨm depolarization: P2Y1, the % diminution in DIOC6(3) MFI and platelet counts were 16.4±3.6 and 14.76±5.78 *vs* 37.61±6.29 and 40.93±7.31, (*p*<0.05, Fig. 8A). For GPIIbIIIa they were: 37.9±1.93 and 74.19±6.54 *vs* 4.39±5.10 and 47.98±2.98 (*p*<0.05, Fig. 8B). As a control, we observed no significant modulation of the cell death percentage when platelets were pre-treated with antagonists of the corresponding platelet receptors in the absence of GD2: MRS2179 100 µM: P2Y1 antagonist; Tirofiban 10 µM: GPIIbIIIa antagonist or DMSO at the same dilution (negative control) (data not shown).

These data suggest that GD2-induced platelet apoptosis is dependent on P2Y1 and GPIIbIIIa receptor cross-linking.

## Discussion

This study describes the first report on the expression and functional role of Siglec-7 in human platelets. Siglec-7 is the most consistently expressed member of the Siglec family proteins in human platelets. Although Siglec-7 is expressed abundantly and exerts a critical role in platelet physiology and apoptosis regulation, it cannot be ruled out that platelets also express other Siglec family proteins with essential functions. The classically accepted description of the function of Siglecs is the detection of the “self” sialome and downregulation of the immune system by expression of ITIM motifs [18], [32].

In this study, we show that human platelets express Siglec-7 and that this expression is increased upon TRAP stimulation. Siglec-7 is localized in the same compartment as specific makers of α-granules (such as CD62P). We observed a close correlation between the expression of CD62P and Siglec-7 on the membrane surface of both resting and TRAP activated platelets. Binding of Siglec-7 to its elective ligand, GD2, did not significantly enhance platelet CD62P expression, aggregation or soluble factor secretion. We observed a similar result with PAC-1 expression. CD62p and PAC-1 are both generally regarded as “benchmark” indicators of platelet activation [33], [34], [35]. Rather, Siglec-7 cross-linking with GD2 increased apoptosis and platelet death by the extra-mitochondrial cellular and mitochondria-dependent (intrinsic) pathways. Specific inhibitors of NAPDH oxidase, PI3K, and PKC, partially but significantly, rescued platelets from apoptosis induced by Siglec-7 engagement; whereas, inhibitors of NFκB had little effect. Furthermore, GD2-induced platelet apoptosis was regulated by the receptors, P2Y1 and GPIIbIIIa. This observation is interesting with regard to platelet physiology, as these receptors are instrumental in primary hemostasis, and the inhibitors used in the current study have previously been shown to dampen hemostasis and thrombosis.

The P2Y1 receptor and fibrinogen receptor might have a role in the effects induced by GD2 as follows: i) P2Y1 receptors or fibrinogen receptors are coreceptors for GD2; or ii) S7 is engaged for the first time (after stimulation by GD2), which activates P2Y1 and/or GPIIbIIIa (inside-out signaling). In both cases, antagonists of these receptors reduce GD2-induced platelet apoptosis. Thus, it is possible that agonists of the P2Y1 receptor and fibrinogen receptor (ADP or fibrinogen) can support the effects of GD2. However, after activation, platelets upregulate Siglec 7 levels, and thus it will be difficult to distinguish whether the effect is induced by the high expression of Siglec 7 or the activation of P2Y1 receptor and fibrinogen receptor. In addition, various platelet activators involved in the secretion of ADP can activate GPIIbIIIa (inside-out). Thus, the binding of ADP and fibrinogen to their corresponding receptors might be involved in the effects induced by GD2.

We observed 36.00±0.84% of CD41a+ platelets expressed Siglec-7 in the absence of stimuli. Thus, GD2 can have an effect on platelets via Siglec-7 even if this receptor is mainly inside the resting platelets, when compared with other gangliosides such as GD3. Martini F et al. [31] described that after platelet activation, GD3 was quickly expressed on the platelet surface and internalized to the cytoskeleton, where it briefly linked with the Src family tyrosine kinase Lyn and the Fc receptor gamma chain. This series of activation steps leads to enhanced CD32 (Fc receptor isoform present in platelets) expression on the platelet membrane. These data suggest that GD3 might act as a second messenger in the activator cascade, which leads to CD32 expression and triggers platelet adhesion and spreading to the subendothelial matrix. Moreover, we could not exclude the fact that our protocol only analyzed individual platelets and that we ignored subpopulations of platelets with different phenotypes (such as Siglec and TLR among others).

This work further reinforces the concept that platelet receptors link the functions of hemostasis and innate immunity [2], [4]. We previously reported the expression of TLRs in human platelets and we and others indicated that TLRs in platelets regulate the secretion of pro-inflammatory cytokines upon exposure to pathogenic microbes [36], [37], [38], [39]. We now describe the expression of Siglec(s) (with particular focus on Siglec-7), which are considered to link hemostasis (this work) and anti-infectious immunity [15], [40]. The CD33-related subset of inhibitory Siglec(s) recognize sialic acid ligands as “self-motifs” and deliver inhibitory signals to innate immune cells. The present study extends this repertoire to platelets. Siglec family members are characterized as dampeners of immune reactivity and promoters of apoptosis. Here we demonstrate that one member of the Siglec family, Siglec-7 is closely associated with platelet apoptosis. We provide evidence that cell loss and platelet death is due to Siglec-7::Ligand-induced apoptosis, and that intrinsic and extrinsic pathways are preferentially involved. This identifies a novel function for platelets in pathology beyond their physiological role, an issue that highlighted when platelets and PMPs were found to infiltrate inflamed tissues and enhance pathology [41], [42]. The present work re-emphasizes and extends the role of platelet receptors in innate immunity and in pathogen sensing.

Even though platelet components stored to make an inventory for transfusion purposes platelets are kept sterile (aseptic) without the acknowledged presence or the deliberate addition of PAMPs, they can generate DAMPs under oxidative stress [43]. Platelet TLR cross-linking by ligands can promote an inflammatory response. Furthermore, Siglec(s) form a complex with CD24, a glycosyl-phosphatidyl-inositol–anchored molecule expressed by immature hematopoietic cells, that recognizes DAMPs and reduces immune system-mediated damage [44]. The platelet expression of Siglec-7 may thus be part of a complex mechanism of auto-regulation of pro-inflammatory platelets.

Previous studies have reported the incidence of accelerated apoptosis during the storage of platelet concentrates [45], [46], [47]. In this regard, a comprehensive knowledge of platelet apoptosis would be beneficial at several levels: first, apoptosis markers could be measured to predict the viability of the given platelet component; second, a means to rescue platelet apoptosis could be used to increase platelet lifespan and recirculation in the transfused patient, and to achieve primary hemostasis [48]. Indeed, the lifespan of platelets during circulation is 6-10 days [49]; thus, there is a significant decrease of platelet viability during 5 day storage [50], [51], [52]. Treatment of diseases such as immune thrombocytopenia requires a target for improving transfused platelet lifespan. Since these are associated with massive apoptosis, the Siglec pathway would be a potential target.

## Supporting Information

File S1 **Supporting information.** Table S1, Monoclonal antibodies used for flow cytometry to analyze the membrane expression of platelets. Figure S1, Representative maximum projection confocal microscopy z series images of Siglec-7 (red) and CD62P (green), demonstrating colocalization (z tack  = 0.6 µm) (five series for each platelet sample: n = 3). Figure S2, Expression of CD33r Siglecs on the membrane surface of unstimulated and TRAP-stimulated platelets analyzed by flow cytometry. A. Representative scattergram from platelet samples of 10 healthy donors. Data expressed as percentage of CD41^+^Siglec^+^ cells. B. Percentage of CD41^+^Siglec^+^ cells. Data expressed as mean ± SEM, (n = 10). *: Significant difference (*t*-test, *p*<0.05) between TRAP-stimulated platelets vs unstimulated platelets. Figure S3, Concentration of soluble CD62P (A) and soluble Siglec-7 (B) in supernatants (n = 10) of resting and TRAP-induced platelets activation over time. Data are shown as pg/2×10^8^ platelets/ml and expressed as mean ± SEM. * Significant differences in sCD62P levels in supernatants of TRAP-stimulated platelets vs unstimulated platelets (*t*-test, *p*<0.05), ¥ and #: significant difference of sCD62P concentration in supernatants over time vs 0 min (ANOVA, *p*<0.05). Figure S4, Concentration of soluble MMP-1 (A), MMP-2 (B), and MMP-9 (C) in supernatants (n = 5) from resting and TRAP-induced platelet activation over time. Data were adjusted to pg/200,000 plot and expressed as mean ± SEM. *significant difference (ANOVA, *p*<0.05) between MMP-1 concentration over time *vs* 0 min. Figure S5, Flow cytometry analysis of CD3, CD14, CD15, CD19, and CD41 expression in platelet preparations. Peripheral blood was collected from healthy donors in endotoxin-free tubes with 3.2% sodium citrate. Platelet-rich plasma (PRP) was prepared by centrifuging the blood at 150 ×*g* for 12 min at 22°C. PRP residual mononuclear cells (A,B) were counted by flow cytometry and compared with peripheral blood (C,D). There was a marked reduction in contaminating cells (CD3-T cells, CD19-B cells, CD15-neutrophils or CD14-monocytes) in platelet preparations in PRP conditions compared with peripheral blood (data are expressed percentage expression (± SD; n = 10 experiments). One representative experiment is shown (A, B).
(DOCX)
