# Supplementary material for: Role of Siglec-7 in Apoptosis in Human Platelets
Source: PLoS One. 2014 Sep 17;9(9):e106239. doi: 10.1371/journal.pone.0106239 (PMC4167548; doi:10.1371/journal.pone.0106239)
Supplement: File S1 — Supporting information. Table S1, Monoclonal antibodies used for flow cytometry to analyze the membrane expression of platelets. Figure S1, Representative maximum projection confocal microscopy z series images of Siglec-7 (red) and CD62P (green), demonstrating colocalization (z tack = 0.6 µm) (five series for each platelet sample: n = 3). Figure S2, Expression of CD33r Siglecs on the membrane surface of unstimulated and TRAP-stimulated platelets analyzed by flow cytometry. A. Representative scattergram from platelet samples of 10 healthy donors. Data expressed as percentage of CD41+Siglec+ cells. B. Percentage of CD41+Siglec+ cells. Data expressed as mean ± SEM, (n = 10). *: Significant difference (t-test, p<0.05) between TRAP-stimulated platelets vs unstimulated platelets. Figure S3, Concentration of soluble CD62P (A) and soluble Siglec-7 (B) in supernatants (n = 10) of resting and TRAP-induced platelets activation over time. Data are shown as pg/2×108 platelets/ml and expressed as mean ± SEM. * Significant differences in sCD62P levels in supernatants of TRAP-stimulated platelets vs unstimulated platelets (t-test, p<0.05), ¥ and #: significant difference of sCD62P concentration in supernatants over time vs 0 min (ANOVA, p<0.05). Figure S4, Concentration of soluble MMP-1 (A), MMP-2 (B), and MMP-9 (C) in supernatants (n = 5) from resting and TRAP-induced platelet activation over time. Data were adjusted to pg/200,000 plot and expressed as mean ± SEM. *significant difference (ANOVA, p<0.05) between MMP-1 concentration over time vs 0 min. Figure S5, Flow cytometry analysis of CD3, CD14, CD15, CD19, and CD41 expression in platelet preparations. Peripheral blood was collected from healthy donors in endotoxin-free tubes with 3.2% sodium citrate. Platelet-rich plasma (PRP) was prepared by centrifuging the blood at 150 ×g for 12 min at 22°C. PRP residual mononuclear cells (A,B) were counted by flow cytometry and compared with peripheral blood (C,D). There was a marked reduc [file pone.0106239.s001.docx]

***Supplemental Materials – Nguyen K.A. et al 2014***

***List of reagents and solutions used in this study***

**Reagents used to stimulate platelets**

Thrombin Receptor Activating Peptide (TRAP), PAR-4 activating peptide, Adenosine 5' -diphosphate sodium salt (ADP), Tyrode’s solution (amount in g/l: NaCl, 8.00; KCl, 0.20; CaCl_2_, 0.20; MgCl_2_, 0.10; NaH_2_PO_4_, 0.05; NaHCO_3_, 1.00; Glucose, 1.00, pH 6.5), LY-294002 hydrochloride, a PI3K inhibitor; BAY-11, an NFκB inhibitor; (S)-(+)-Clopidogrel hydrogen sulfate, a P2Y12 antagonist; MRS 2179 ammonium salt hydrate, a P2Y1 antagonist; and Tirofiban hydrochloride monohydrate, a GPIIbIIIa antagonist were purchased from Sigma Aldrich (Saint-Quentin Fallavier, France). Collagen was purchased from Nycomed (Paris, France); Bis-indolylmaleimide I (BIM I), a PKC inhibitor; SCH 79797 dihydrochloride, a PAR-1 antagonist; *trans*-Cinnamoyl-Tyr-Pro-Gly-Lys-Phe-amide trifluoroacetate salt (tcY-NH2), a PAR-4 antagonist were from R&D Systems (R&D Systems, Europe, Lille, France). Gangliosides, GD2, GD3, GT1b, Diphenyleneiodonium Chloride (DPI), and A23187 were from Calbiochem (Merck Millipore, Guyancourt, France). Phosphate-buffered saline (PBS) was from PAA (Les Mureaux, France).

***Preparation of platelet-rich plasma and platelet stimulation***

Peripheral blood samples from healthy subjects were collected in endotoxin-free tubes with 3.2% sodium citrate (Vacutainer^®^, Becton Dickinson, San Jose, California) [[1](#_ENREF_1)]. Informed consent of all donors was obtained prior to blood collection by the Regional Blood Service. The blood samples were centrifuged at 192 ×*g* for 10 min at room temperature (RT) to obtain platelet-rich plasma (PRP). Platelet counts were determined using a Coulter LH500 (Beckman-Coulter, Villepinte, France).

***Analysis of endotoxin levels in platelet concentrates***

PRPs were examined for bacterial contamination by conventional hemoculture techniques and endotoxin levels were measured using a *Limulus amoebocyte* lysate kit (QCL-1000) according to the manufacturer’s instructions (Cambrex Bio Science, Walkersville, MD), (Supplemental material) [[1](#_ENREF_1),[2](#_ENREF_2)]. Briefly, following 10 min incubation at 70°C, platelets were diluted in pyrogen-free water (1:20), and incubated with *L. amoebocyte* lysate at 37°C. After 10 min, chromogen was added and samples were incubated for an additional 6 min, and the absorbance at 405 nm was measured. Endotoxin levels were expressed in international units (IU) per mL [[1](#_ENREF_1),[2](#_ENREF_2)].

***Platelet marker analysis***

PRPs were diluted with 1×PBS to a final concentration of 5×10^6^ platelets/100 µl. Platelet suspensions were incubated with the appropriate monoclonal antibody (mAb) for 30 min at RT in the dark, and washed once with 1×PBS. The source of reagents is quoted in the reagent list (**Table: S1**). As all platelets constitutively express CD41a, this marker conjugated to fluorescein isothiocyanate (5 µL/test) was used to define gates for subsequent experiments. Activated platelets are characterized by their expression, among other markers, of CD62P and PAC-1. APC- or PE-conjugated mAbs against human CD62P were used. Flow cytometry was performed on a FACSvantage SE device equipped with CellQuestS-Pro software (BD-Biosciences) [[1](#_ENREF_1)].

Cytosolic proteins were quantified by Bradford assay (Coomassie Blue) purchased from Sigma Aldrich, Saint-Quentin Fallavier, France. Equal amounts of proteins were separated on 9% sodium dodecyl sulfate polyacrylamide gel electrophoresis with 1×Laemmli Sample Buffer (Sigma Aldrich); human cell lysates, Hela (epitheloid carcinoma cells) and HuT-78 (T lymphocyte lymphoma cells) were used as positive controls for Bak and Bax, respectively. Following transfer to cellulose membranes (Healthcare, Vélizy, France), the membrane was stained with rabbit anti-Bax and anti-Bak primary mAbs (Santa Cruz Biotechnology, Yvelines, France) and peroxidase conjugated-goat anti-rabbit secondary antibody (whole molecule; Sigma Aldrich, Saint-Quentin Fallavier, France). The signal from secondary antibody was detected using an enhanced chemiluminescence system (Sigma Aldrich). Peroxidase-conjugated mouse anti-actin mAb (Sigma Aldrich) was used as an internal loading control.

***Quantification of platelet soluble factors***

sCD62P, MMP-1, 2, 9, sSiglec-7 and serotonin content in platelet supernatants were quantified using commercial ELISA kits (purchased from R&D Systems Europe Ltd., Lille, France), except for the serotonin ELISA (IBL International, Hamburg, Germany). Absorbance at 450 nm (for serotonin, 405 nm) was measured using an ELISA plate reader (Magellan Software, Sunrise TM, Tecan Group Ltd, Lyon, France). Results were normalized to 2×10^8^ platelets/ml [[1](#_ENREF_1)].

The sCD40L and RANTES content in platelet supernatants was quantified using Luminex™ technology (using a human cytokine/chemokine magnetic bead panel I, HCYTOMAG-60K, Millipore, Molsheim, France) following the manufacturer’s instructions. Results were acquired with the Bioplex-200 system (Bioplex-Manager™ software, Biorad, Marnes-la-Coquette, France) and adjusted to pg/2×10^8^ platelets/ml.

***Determination of platelet aggregation***

The aggregometer was calibrated using PRP (0% light transmission, 100% aggregation) and platelet poor plasma (PPP, 100% light transmission, 0% aggregation). The PPP was obtained by centrifugation of PRP at 3000 ×*g* for 20 min at RT. Samples were treated with Siglec-7 ligand, GD2 (5 µg/ml) or vehicle control for 30 min, at 37°C, with constant stirring, followed by stimulation with ADP (10 µM). Platelet aggregation was monitored by the Thrombo-aggregometer at 4V using Thrombosoft 1.6 software (SD Medical Throuard, France).

***Platelet marker analysis by confocal microscopy***

*Protein staining*

Unless otherwise stated, all mAbs were purchased from Abcam (Paris, France). Immunostaining was performed following previously described protocols [[3](#_ENREF_3)]. In brief, 100 µl of unstimulated platelets in PRP were fixed with 4% paraformaldehyde (PFA), permeabilized with 0.5% Triton X-100, washed twice and blocked with 2% bovine serum albumin in PBS for 20 min. Platelets were resuspended in 100 µl of 1×PBS and incubated with rabbit anti-human Siglec-7 along with one of the following marker antibodies: mouse anti-human CD62P mAb (clone Psel.KO.2.5) for granules; serotonin (clone 5HT-H209) for dense granules; LAMP-1 (clone FMC46) for lysosomes; M6P (clone MEM-238) for endosomes and tubulin (clone TU-01) for cytoskeleton. Following incubation, primary antibodies were washed and platelets were incubated with Alexa Fluor 555 conjugated-goat anti rabbit IgG and Alexa Fluor 488 conjugated goat-anti mouse IgG (Invitrogen, Saint Aubin, France). For co-staining with CD41a and Siglec-7, following primary and secondary antibody staining against Siglec-7, platelets were incubated with Alexa 488-conjugated CD41a. For co-staining with CD62P and Siglec-7, platelets were initially incubated with a CD62P mAb, followed by a biotinylated anti-mouse IgG (whole molecule, Sigma), and with Streptavidin/Alexa Fluor 488 conjugate (Invitrogen) then antibodies to Siglec-7. Platelets were washed three times and allowed to adhere to polylysine-coated cover slips (Labomoderne, Paris, France) at 37°C for 20 min, and fixed in 4% PFA for 10 min. The cover slips were washed twice and mounted using Vectashield mounting solution (Vector Laboratories, Clinisciences Nanterre, France).

*Confocal microscopy: image acquisition and analyses*

Samples were examined with a Leica TCS-SP2 confocal scanning laser inverted microscope (Leica-Microsystem, Heidelberg, Germany). Fluorescence images of two or three colors were captured sequentially using a set of three continuous wave lasers delivering monochromatic light at wavelength equal to 488 (for Alexa Fluor 488), 543 (for Alexa Fluor 555) and 633 nm (for Alexa Fluor 647). For each sample, a series of x-y images were collected along the z-axis at 0.3 μm intervals using an oil immersion objective (HCX PL APO 63X 1.4NA) and an optical zoom of 2× or 4×. Image stacks were analyzed and processed using ImageJ software [[3](#_ENREF_3)]. Colocalization analyses were performed for stacks and merged images. Pearson’s correlation coefficient values were calculated using the Intensity Correlation Analysis plugin [[4](#_ENREF_4)]; 2D intensity scatter plots were generated using the colocalization threshold macro and frequency scatter plots were obtained with the “red-green correlator” plugin. Representative data from a stack of five images for each labeling condition were shown.

*Analyses of intracellular compartments*

Platelets were treated with the following inhibitors or the appropriate vehicle controls: LY-294002 hydrochloride (PI3K inhibitor [[5](#_ENREF_5)]; 50 µM; 30 min at 37°C); BAY 11-7082 (an NFκB inhibitor (IKK) [[6](#_ENREF_6)]; 5 µM; 1 h at RT); bisindolylmaleimide I (BIM I, a PKC inhibitor [[7](#_ENREF_7)]; 50 µM; 15 min at 37°C); diphenyleneiodonium chloride (DPI, an NAPDH oxidase inhibitor [[8](#_ENREF_8)];1 µM; 30 min at 37°C); (S)-(+)-clopidogrel hydrogen sulfate (a P2Y12 antagonist [[9](#_ENREF_9)]; 400 µM; 30 min at 37°C); MRS 2179 ammonium salt hydrate (a P2Y1 antagonist [[9](#_ENREF_9)]; 100 µM; 30 min RT); tirofiban hydrochloride monohydrate (a GPIIbIIIa antagonist [[10](#_ENREF_10)];10 µM; 15 min at 37°C); SCH 79797 dihydrochloride (a PAR-1 antagonist [[11](#_ENREF_11)]; 10 µM; 15 min at 37°C); and *trans*-cinnamoyl-Tyr-Pro-Gly-Lys-Phe-amide trifluoroacetate salt (tcY-NH2; a PAR-4 antagonist [[12](#_ENREF_12)]; 400 µM; 15 min at 37°C). Following treatment, platelets were stimulated with GD2 (as described in an earlier section) and analyzed for apoptosis markers.

***References***

1. Cognasse F, Hamzeh-Cognasse H, Lafarge S, Delezay O, Pozzetto B, et al. (2008) Toll-like receptor 4 ligand can differentially modulate the release of cytokines by human platelets. Br J Haematol 141: 84-91.

2. Hamzeh-Cognasse H, Cognasse F, Palle S, Chavarin P, Olivier T, et al. (2008) Direct contact of platelets and their released products exert different effects on human dendritic cell maturation. BMC Immunol 9: 54-69.

3. Thon JN, Peters CG, Machlus KR, Aslam R, Rowley J, et al. (2012) T granules in human platelets function in TLR9 organization and signaling. The Journal of cell biology 198: 561-574.

4. Li Q, Lau A, Morris TJ, Guo L, Fordyce CB, et al. (2004) A syntaxin 1, Galpha(o), and N-type calcium channel complex at a presynaptic nerve terminal: analysis by quantitative immunocolocalization. J Neurosci 24: 4070-4081.

5. Zhang S, Ye J, Zhang Y, Xu X, Liu J, et al. (2013) P2Y12 protects platelets from apoptosis via PI3k-dependent Bak/Bax inactivation. J Thromb Haemost 11: 149-160.

6. Lee J, Rhee MH, Kim E, Cho JY (2012) BAY 11-7082 is a broad-spectrum inhibitor with anti-inflammatory activity against multiple targets. Mediators Inflamm 2012: 416036.

7. Harper MT, Poole AW (2011) PKC inhibition markedly enhances Ca2+ signaling and phosphatidylserine exposure downstream of protease-activated receptor-1 but not protease-activated receptor-4 in human platelets. J Thromb Haemost 9: 1599-1607.

8. Choo HJ, Saafir TB, Mkumba L, Wagner MB, Jobe SM (2012) Mitochondrial calcium and reactive oxygen species regulate agonist-initiated platelet phosphatidylserine exposure. Arterioscler Thromb Vasc Biol 32: 2946-2955.

9. Labarthe B, Babin J, Bryckaert M, Theroux P, Bonnefoy A (2012) Effects of P2Y(1) receptor antagonism on the reactivity of platelets from patients with stable coronary artery disease using aspirin and clopidogrel. Br J Pharmacol 166: 221-231.

10. Leytin V, Mutlu A, Mykhaylov S, Allen DJ, Gyulkhandanyan AV, et al. (2009) The GPIIbIIIa antagonist drugs eptifibatide and tirofiban do not induce activation of apoptosis executioner caspase-3 in resting platelets but inhibit caspase-3 activation in platelets stimulated with thrombin or calcium ionophore A23187. Haematologica 94: 1783-1784.

11. Lee H, Hamilton JR (2013) The PAR1 antagonist, SCH79797, alters platelet morphology and function independently of PARs. Thromb Haemost 109: 164-167.

12. Ma L, Perini R, McKnight W, Dicay M, Klein A, et al. (2005) Proteinase-activated receptors 1 and 4 counter-regulate endostatin and VEGF release from human platelets. Proc Natl Acad Sci U S A 102: 216-220.

**Supplemental figures – Nguyen K.A. et al 2014**

| *Name* | *Clone* | *Format* | *Isotype* | *Brand* |
| --- | --- | --- | --- | --- |
| CD41a | **HIP8** | **FITC** | **Ms IgG1, k** | **BD BIOSCIENCES** |
| CD62P | **AK-4** | **APC/PE** | **Ms IgG1, k** | **BD BIOSCIENCES** |
| CD63 | **H5C6** | **PE** | **Ms IgG1, k** | **BD BIOSCIENCES** |
| CD40L | **TRAP1** | **APC** | **Ms IgG1, k** | **BD BIOSCIENCES** |
| SIGLEC-7 | **194212** | **ALEXA FLUOR 488** | **IgG2B** | **R&D SYSTEMS** |
| SIGLEC-9 | **191240** | **PE** | **IgG2A** | **R&D SYSTEMS** |
| SIGLEC-5/14 | **194128** | **PE** | **IgG1** | **R&D SYSTEMS** |
| SIGLEC-10 | **pAb** | **PE** | **IgG** | **R&D SYSTEMS** |
| SIGLEC-11 | **pAb** | **PE** | **IgG** | **R&D SYSTEMS** |
| TRAIL R1 | **69036** | **PE** | **IgG1** | **R&D SYSTEMS** |
| ANNEXIN V |  | **APC** |  | **MABTAG** |
| ISOTYPE CONTROL | **MOP-21** | **FITC/APC/Alexa Fluor 488 / PE** | **Ms IgG1, k** | **BD BIOSCIENCES** |

**Table S1.** Monoclonal antibodies used for flow cytometry to analyze the membrane expression of platelets.


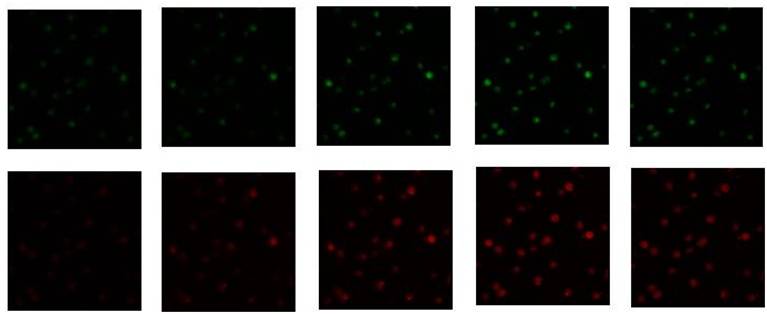


**Figure S1.** Representative maximum projection confocal microscopy z series images of Siglec-7 (red) and CD62P (green), demonstrating colocalization (z tack = 0.6 μm) (five series for each platelet sample: n=3).


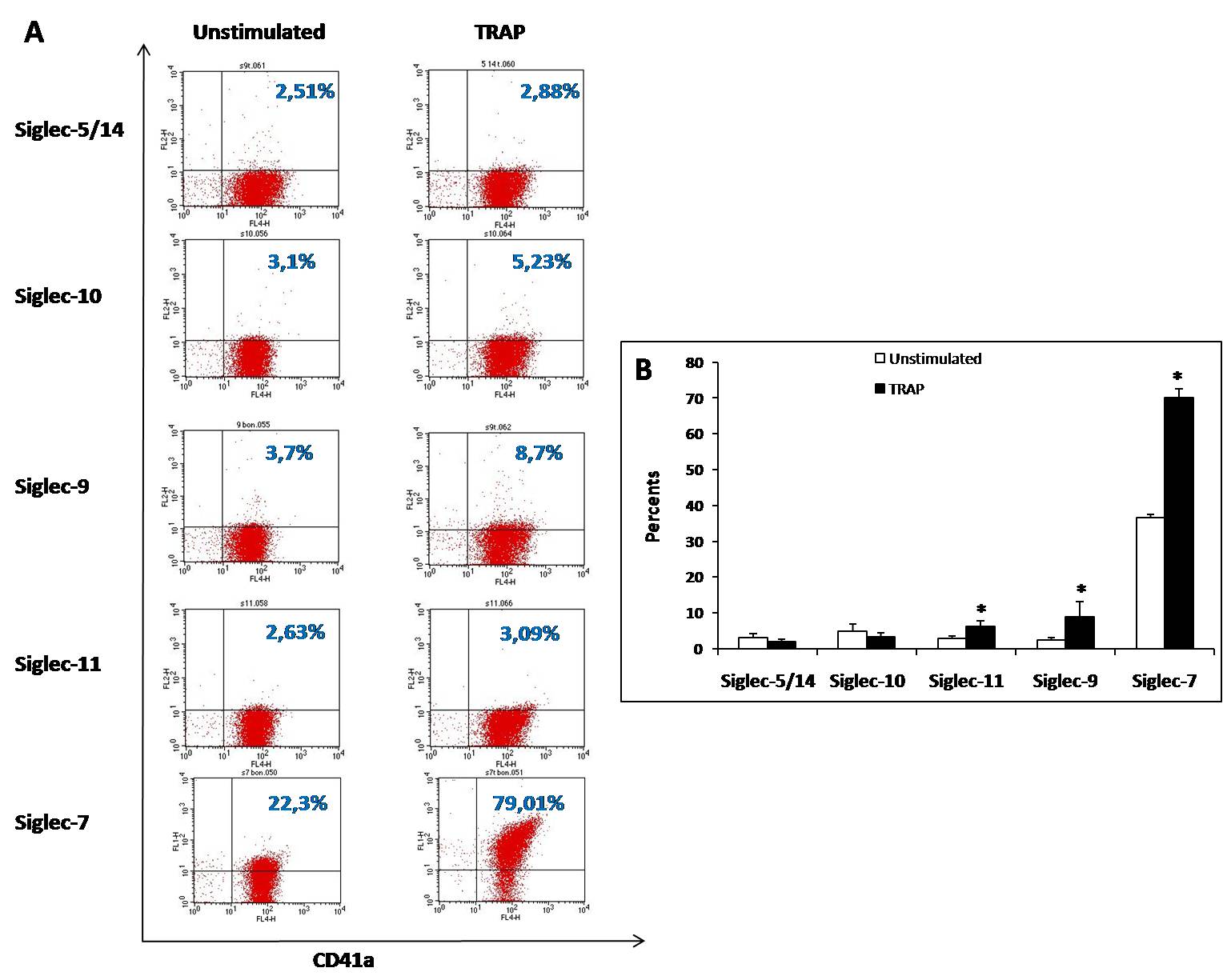


**Figure S2.** Expression of CD33r Siglecs on the membrane surface of unstimulated and TRAP-stimulated platelets analyzed by flow cytometry. **A.** Representative scattergram from platelet samples of 10 healthy donors. Data expressed as percentage of CD41^+^Siglec^+^ cells. **B.** Percentage of CD41^+^Siglec^+^ cells. Data expressed as mean ± SEM, (n=10). *****: Significant difference (*t*-test, *p*<0.05) between TRAP-stimulated platelets vs unstimulated platelets.

**
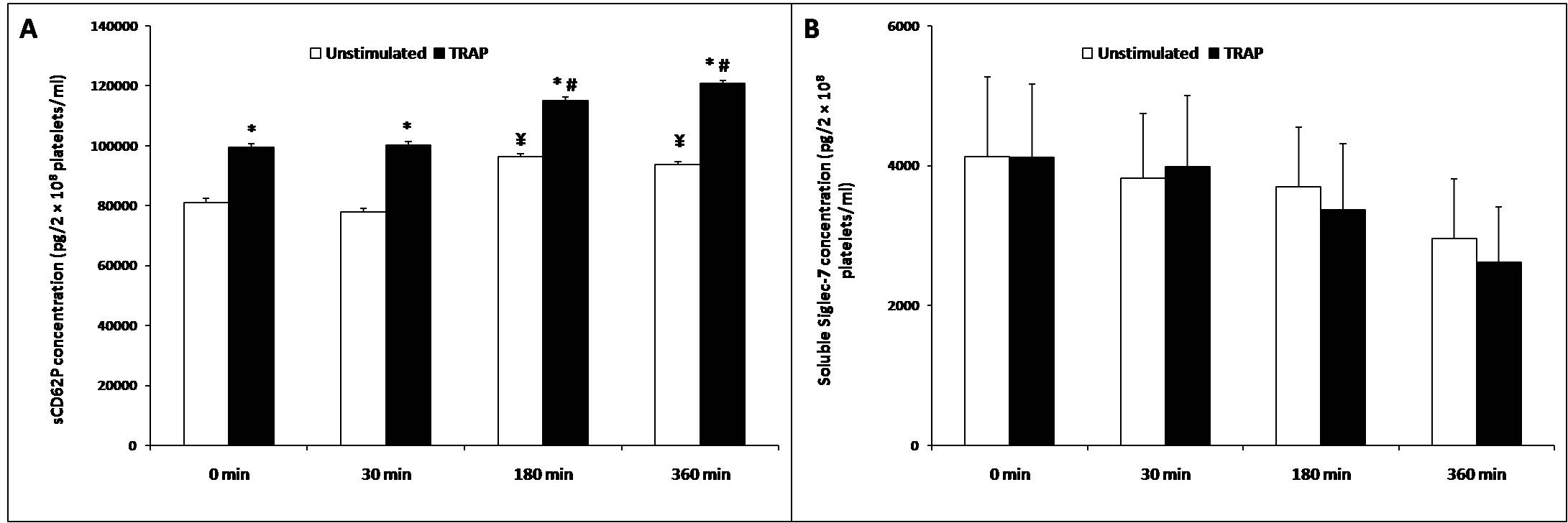
**

**Figure S3.** Concentration of soluble CD62P (A) and soluble Siglec-7 (B) in supernatants (n=10) of resting and TRAP-induced platelets activation over time. Data are shown as pg/2×10^8^ platelets/ml and expressed as mean ± SEM. * Significant differences in sCD62P levels in supernatants of TRAP-stimulated platelets vs unstimulated platelets (*t*-test, *p*<0.05), ¥ and #: significant difference of sCD62P concentration in supernatants over time vs 0 min (ANOVA, *p*<0.05).

**
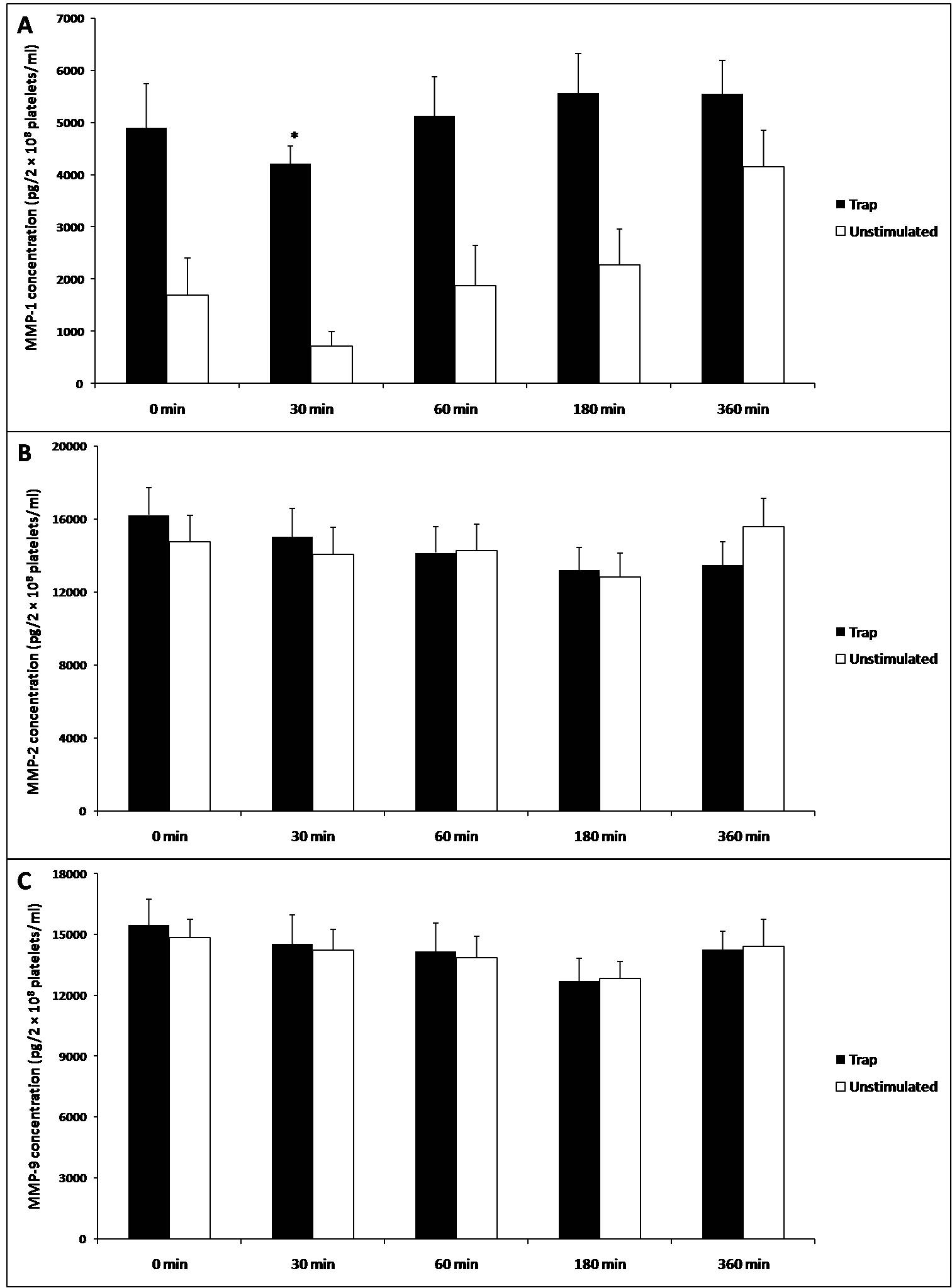
**

**Figure S4.** Concentration of soluble MMP-1 (A), MMP-2 (B), and MMP-9 (C) in supernatants (n=5) from resting and TRAP-induced platelet activation over time. Data were adjusted to pg/200,000 plot and expressed as mean ± SEM. *significant difference (ANOVA, *p*<0.05) between MMP-1 concentration over time *vs* 0 min.


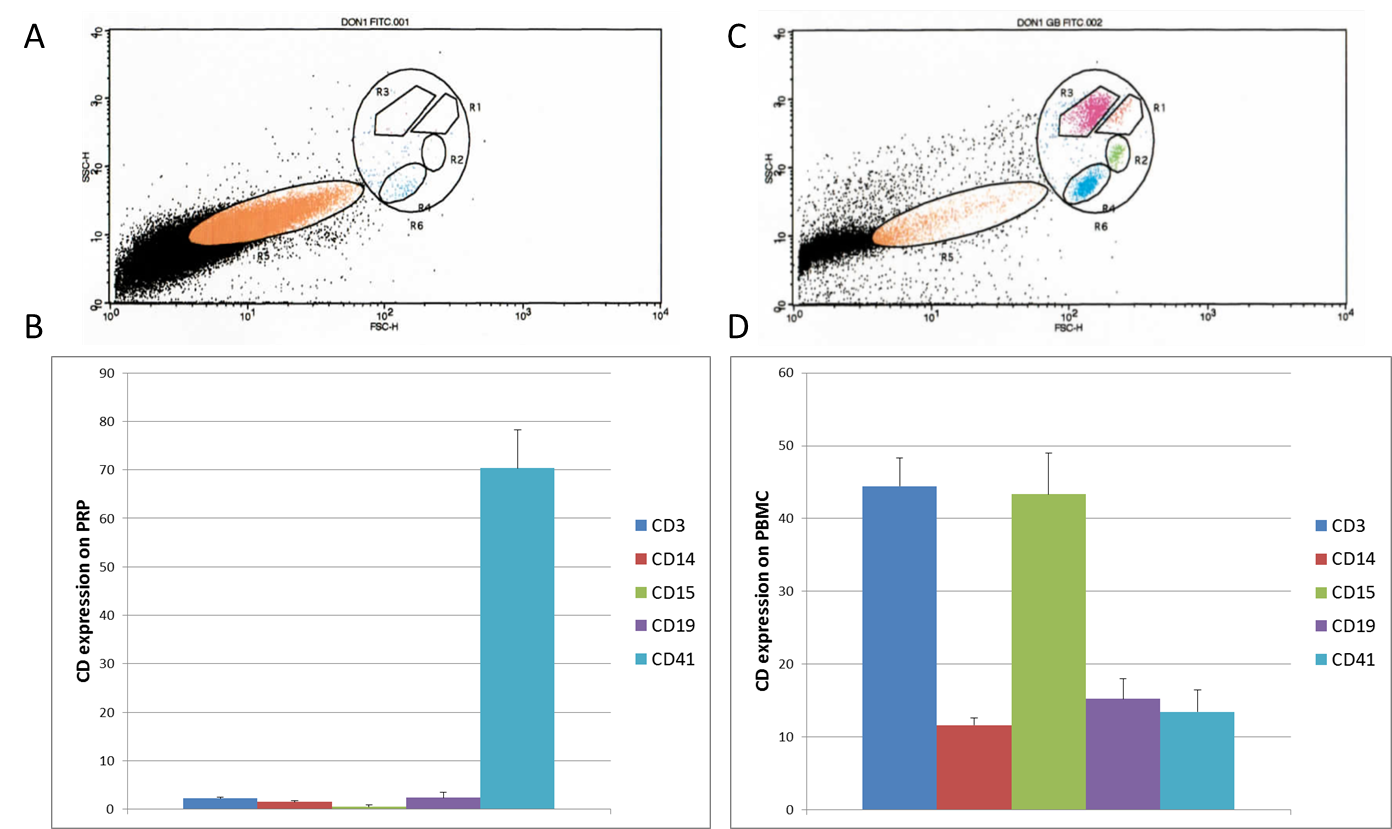


**Figure S5.** Flow cytometry analysis of CD3, CD14, CD15, CD19, and CD41 expression in platelet preparations. Peripheral blood was collected from healthy donors in endotoxin-free tubes with 3.2% sodium citrate. Platelet-rich plasma (PRP) was prepared by centrifuging the blood at 150 ×*g* for 12 min at 22°C. PRP residual mononuclear cells (A,B) were counted by flow cytometry and compared with peripheral blood (C,D). There was a marked reduction in contaminating cells (CD3-T cells, CD19-B cells, CD15-neutrophils or CD14-monocytes) in platelet preparations in PRP conditions compared with peripheral blood (data are expressed percentage expression (± SD; n=10 experiments). One representative experiment is shown (A, B).
